# Phylogenetic and genetic evolutionary analyses of the mitochondrial genome of *Mastophorus muris* in *Neodon fuscus* from the Qinghai-Tibetan Plateau

**DOI:** 10.1128/spectrum.03067-24

**Published:** 2025-07-07

**Authors:** Hongrun Ge, Haining Zhang, Ru Meng, Shengrui Xu, Hailong Zhao, Weishan Lin, Jing Li, Yong Fu

**Affiliations:** 1 Academy of Animal Sciences and Veterinary Medicine, State Key Laboratory for Diagnosis and Treatment of Severe Zoonotic Infectious Diseases, Key Laboratory for Zoonosis Research of the Ministry of Education, Basic Medicine Department of Medical College, Qinghai University 207475, Xining, Qinghai, Republic of China; 2 Qinghai Provincial Key Laboratory of Pathogen Diagnosis for Animal Diseases and Green Technical Research for Prevention and Control, Xining, Republic of China; 3 Xining Animal Disease Control Center, Xining, Republic of China; 4 Animal Husbandry and Veterinary Station of Huangyuan County, Xining, Republic of China; 5 Animal Disease Prevention and Control Center of Qinghai Province, Xining, Republic of China; Hubei University of Medicine, Shiyan, China

**Keywords:** *Mastophorus muris*, mitochondrial DNA, phylogenetic analysis, divergence time

## Abstract

The larvae of the nematode family can infect several animals. *Mastophorus muris* is a parasitic nematode that infects mammals and has a worldwide distribution. In this study, the phylogenetic relationships and differentiation time of *M. muris* collected from *Neodon fuscus* were studied using molecular methods. The phylogenetic tree constructed based on 12 protein-coding genes (PCGs), cytochrome oxidase subunit I (*cox 1*), 18S rRNA, and 28S rRNA showed that *M. muris* was closely related to the Onchocercidae family and that this relationship was highly node-supported. The mitochondrial genome of *M. muris* was found to be a circular double-stranded DNA molecule with 32 mitochondrial genes, including 12 PCGs (ND4L, CYTB, ND1-6, *cox 1-3*, ATP6), 18 tRNA genes, and 2 rRNA genes (s-rRNA and L-rRNA). The divergence time based on mitochondrial *cox 1* generation suggests that *M. muris* was formed approximately 391 million years ago. Overall, this study can provide valuable data for the further study of the phylogeny and differentiation time of *M. muris* in the Qinghai-Tibetan Plateau.

**IMPORTANCE:**

Our study demonstrates the close genetic relationship between *M. muris* and the Onchocercidae family. Although previous studies have demonstrated this phylogenetic relationship, it was confirmed in our study using the genetic markers *cox 1*, 18S rRNA, 28S rRNA, and the 12 protein-coding genes of *M. muris* parasitized on *Neodon fuscus* from the Qinghai-Tibetan Plateau (QTP). A relatively close relationship was also found between *M. muris* and the Tetrameridae family. We analyzed the divergence time of *M. muris* and speculated that *M. muris* in the QTP might have originated in the Mediterranean region and that there might have been a mutual transmission between *Meles meles* in the Mediterranean, transmitting *M. muris* to the QTP.

## INTRODUCTION

*Mastophorus muris* is a cosmopolitan Spirurid nematode parasitizing the stomach of small rodents (1). The intermediate hosts of *M. muris* are arthropods, including Orthoptera: *Locusta migratoria* L., Reticoptera: *Periplaneta americana* L., and Dermaptera: *Labidura riparia* Pallas (2), and also beetles and fleas (3, 4). Previous studies have reported various small rodents as definitive hosts (5, 6). Many countries and islands, including Nigeria (7), Iran (8, 9), Malaysia (10), and El Hierro (11), have conducted studies on *M. muris*.

In the past, the biological identification of *M. muris* was focused on morphological studies (2, 11, 12). Various regions of the internal transcriptional spacer (ITS) and mitochondrial DNA (mtDNA) have received the most attention in the study of the phylogeny of a species (13, 14). MtDNA is a closed, circular, double-stranded DNA that follows strict maternal inheritance (15). It is a valuable source of parasite taxonomy, population genetics, and systematics research (16). The unique characteristics of mtDNA make it an obvious tool for the molecular diagnosis of nematodes, as it is typically present in hundreds of copies per cell; thus, mtDNA can be obtained in preparative yields (17). Studies have shown that mtDNA evolves much faster than ITS and that it is more effective in distinguishing between closely related species (18). MtDNA accumulates substitutions more quickly than ITS, and the difference is more pronounced in the most closely related species pairs (19). Therefore, mtDNA might be the best choice for applications that use sequence data from a small number of individuals to identify potentially cryptic species.

The taxonomy within the *Mastophorus* genus and the diversity in this genus are controversial among taxonomists, and different morphological features have been proposed for this classification (12). More powerful molecular markers are needed to study the phylogenetic relationship between *M. muris* and its location in this subfamily of nematodes. Therefore, mtDNA was chosen as a molecular marker in this study. A recent study has suggested the use of multiple genetic markers to reduce primer bias and improve classification resolution (20). Therefore, the mitochondrial cytochrome *c* oxidase subunit I (*cox 1*), nuclear small ribosomal RNA (18Sr RNA), and large ribosomal RNA (28Sr RNA) genes were selected as genetic markers. To date, *cox 1* has been widely used in mitochondrial gene analysis to study species diversity, diagnosis, and population variation (21). However, there are few studies regarding the use of *cox 1* in studying *M. muris*.

The Qinghai-Tibetan Plateau (QTP) is the world’s loftiest geographic region that significantly influences the regional climate, water resources, ecological surroundings, and human endeavors in the adjacent areas (22). The QTP has the highest biodiversity for an extreme environment worldwide and provides an ideal natural laboratory to study adaptive evolution (23). This region represents a unique ecological niche with diverse wildlife harboring several human pathogens and numerous previously uncharacterized pathogens (24). *Neodon fuscus,* a unique species prevalent in and around the QTP, provides an ideal environment for *M. muris* (25). The eggs invade through the mouthparts of the insect and develop into larvae in the mesentery of the insect. All larvae are encapsulated in blood or adipose tissues, and the adult worms live in the stomachs of small rodents (2). The purpose of studying *M. muris* is to provide a further scientific basis for the epidemiological investigation of *M. muris* in the QTP.

Our study was carried out on the *M. muris* of a small, wild mammal, *Neodon fuscus*, in the Qinghai province. The complete genome of *M. muris* was sequenced and assembled for the first time, and its genome structure and amino acid composition were compared and analyzed. A comprehensive molecular phylogenetic analysis of *M. muris* was undertaken. The findings of this study can expand our understanding of the mitochondrial genome diversity and the phylogenetic relationship of *M. muris* in *Neodon fuscus*. Moreover, this study can provide valuable data to support further research on the phylogenetics and divergence timing of *M. muris*.

## MATERIALS AND METHODS

### Sample source and mitochondrial genome sequencing

Nematode samples were obtained from Gande County, Golog Tibetan Autonomous Prefecture, Qinghai Province (34°00′N; 100°09′E; average altitude at 4,011 m), the People’s Republic of China in 2023. All nematode samples were washed with 1 × phosphate buffer mixed with normal saline, and nematodes were placed in 75% ethanol for molecular and morphological identification.

### DNA extraction, amplification, cloning, and sequencing of marker genes

Commercially available kits (Cat. DP304-03, TIANamp Genomic DNA kit, TIANGEN Biotechnology, Beijing, China) were used to extract genomic DNA from samples. DNA was stored at –20°C until used. *cox 1*, 18S rRNA, and 28S rRNA were amplified using polymerase chain reaction (PCR). Primer information is detailed in Table 1, and PCR conditions are shown in Table S1. The primers were synthesized by Sangon Biotech (Shanghai, China). The extracted DNA was amplified and sequenced following the manufacturer’s instructions. The DNA fragments were amplified using a standard PCR at a final volume of 25 µL. The *cox 1*, 18S rRNA, and 28S rRNA fragments of the sample were amplified and sequenced for further phylogenetic analyses. The PCR products were purified using a TIANgel Midi purification kit (Cat. DP209-02, Tiangen, China). Final PCR products were sent to Sangon Biotech (Shanghai) Co. Ltd. for library construction and sequencing.

**TABLE 1:** PCR primers used in this study

Primer name	Primer sequence (5′−3′)
*cox 1*-2575	TTTTTTGGGCATCCTGAGGTTTAT
*cox 1*-3021	TAAAGAAAGAACATAATGAAAATG
18S-F	GTAGTTATATGCTTGTCTC
18S-R	GCATCACAGACCTGTTATTGCTC
28S-F	CCCCCTGAATTTAAGCATAT
28S-R	GTTAGACTCCTTGGTCCGTG

### Quality control

The quality of the raw data obtained by sequencing was evaluated and filtered, and valid read statistics were carried out using fastp software. Low-quality sequences with linkers in the reads were removed, and relatively accurate clean data were obtained. The sequencing data were statistically analyzed, including Q10, Q20, Q30, and Q40 (Table 2).

**TABLE 2:** Introduction of quality value

Phred quality score	Base call accuracy
Q10	90%
Q20	99%
Q30	99.90%
Q40	99.99%

### Mitochondrial genome assembly and annotation

Whole-genome shotgun strategy was used to construct a library, and next-generation sequencing was used to obtain the mitochondrial genome sequence of *M. muris*. Sequencing was performed on the Illumina HiSeq platform, using paired-end sequencing to sequence the quality-checked fragments. FASTP v0.36 (26) was used to remove low-quality sequences from the original sequences and obtain a clean data set. SPAdes v3.15 (27) was used to splice and assemble the short fragment sequences (clean reads) from high-throughput sequencing. The mitochondrial genome sequence of *M. muris* was obtained using PrInSeS-G for sequence correction to correct the base errors and indels of small fragments in the splicing process. Mitochondrial genome annotation results for *M. muris* were exported using SnapGene v7.0, and a circular map of the mitochondrial genome was plotted. MEGA v11 (28) was used to calculate the codon usage frequency of each protein-coding gene (PCG) in the mitochondrial genome of *M. muris*.

### Phylogenetic analyses

To determine the phylogenetic status of *M. muris* and further analyze its internal phylogenetic relationships, genome sequences were obtained from the following genes: *cox 1*, 18S rRNA, 28S rRNA, and 12 PCGs. The gene sequences obtained from sequencing were compared with those already in GenBank using BLAST. 12 PCGs, 18S rRNA, 28S rRNA, and *cox 1* sequences from other nematode species (new or from GenBank) were used as ingroups. Six families were selected for phylogenetic analysis of the whole-genome sequence (see Table S1 for details), and sequence alignment was achieved using MAFFT v7.505 (29) and the automatic option. trimAI v1.2 was used to trim the alignment sequence using the automatic option (30). IQ-TREE v2.2.0 was used to construct the phylogenetic tree of *M. muris* using the maximum likelihood method (31) and Bayesian inference, and the sequences of *Aglenchus agricola* were used as outgroups. Model selection was performed automatically using ModelFinder (32). All other parameters are set to default. The online tool tvBOT (33) was used to view and modify the phylogenetic tree.

### Analysis of divergence times

The phylogenetic tree was used as a reference for species selection for the analysis of differentiation time. Six species of *Steinernema* (*S. arenarium, S. surkhetense, S. feltiae, S. abbasi, S. sangi,* and *S. carpocapsae*) were selected for divergence time analysis. The divergence time was determined using BEAST v2.7.4 (34), and the clock model was set up to relax uncorrelated log-normal values. The strict clock model was chosen to ignore the rate differences between the branches in the mode. The gamma class count was set to 4, and the GTR alternative model was selected. Tree priori was performed using the calibrated Yule model. Based on previous studies and discussions on the divergence time of the Spirocercidae family, the average of the existing normal distribution of the Spirocercidae and Steinernematidae families was set to 391 million years ago (Mya) (35). Samples from the posterior were drawn every 1,000 steps over a total of 10,000,000 steps per the MCMC run. Other options were run using their default values. Tracer v1.7.2 was used to determine whether the results converged. TreeAnnotator v2.1.2 was used to annotate the trees using maximum clade credibility and median height settings with a 10% burn-in.

## RESULTS

### Morphological observation

**Fig 1:**
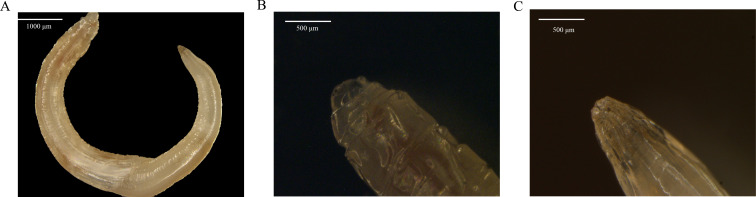
The morphological observation of *M. muris* (**A**). A complete *M. muris*. Bar = 1,000 µm. (**B, C**) Enlarged views of the head and tail respectively. Bar = 500 µm.

The results of the stereomicroscope revealed that the overall length of *M. muris* was approximately 9–10 mm, with a tightly stacked circular head forming a ladder-like appearance and a smooth stratum corneum. There were horizontal stripes on the body. The tail was curled and shaped a corncob-like shape, with longitudinal grooves on the surface. Morphological findings are shown in Fig. 1.

### Distribution of sequencing quality for *M. muris*

**Fig 2:**
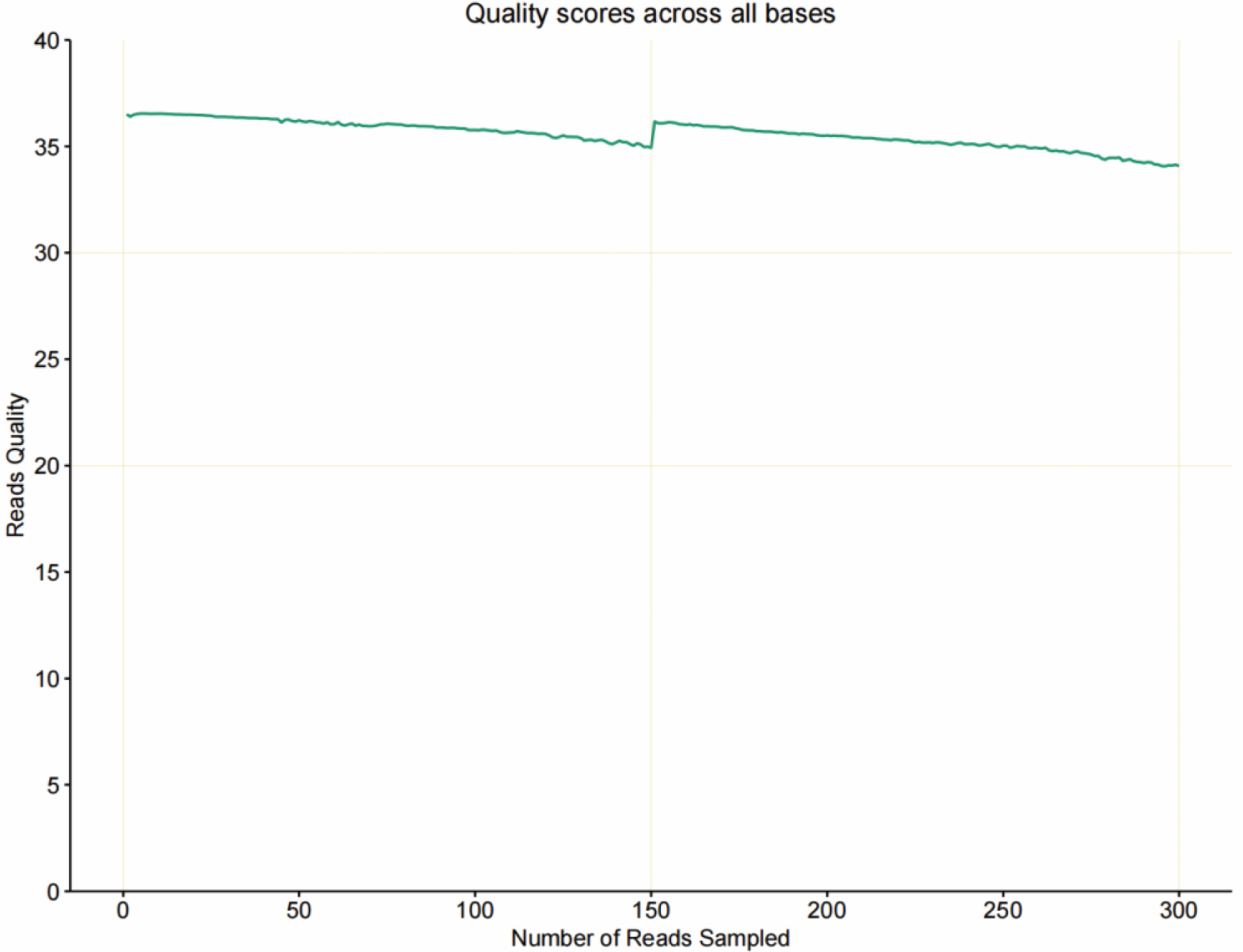
Distribution of sequencing quality for *M. muris*.

Base quality-distribution checks were conducted to detect abnormally high error rates at certain base locations over the length of the sequencing range, e.g., if the base sequencing–error rate in the middle was significantly higher than that in other locations. In this study, the quality of sequencing data was mainly distributed above Q20 (Fig. 2), and the sequencing quality was high.

### Sequence similarity comparison

Results from BLAST alignment indicated that the similarities between *cox 1*, 18S rRNA, 28S rRNA, and 12 PCGs and the known *M. muris* sequences in GenBank were 91.99%–99.21%, 97.82%–98.79%, 93.48%, and 99.14%, respectively (Table 3).

**TABLE 3:** The similarities between the newly obtained sequences of the four *M. muris* genetic markersgenetic markers with those previously deposited in GenBank

Genetic marker	Comparison result with sequences in GenBank		
	Species	Accession number in GenBank	Similarity/%(Average/%)
*cox 1*	*M. muris*	OR262213, MK867480, MG821081, MG386206	91.99–99.21 (96.86)
18S rRNA	*M. muris*	MG818763, OR345241, MN086286–MN086290	97.82–98.79 (98.10)
28S rRNA	*M. muris*	MG818763	93.48
	*Protospirura muricola*	KP760405	89.51
12PCGs	*M. muris*	OR262213	99.14
	*Setaria digitata*	NC014284	81.04

### Mitochondrial genome protein-coding genes and codon usage

**Fig 3:**
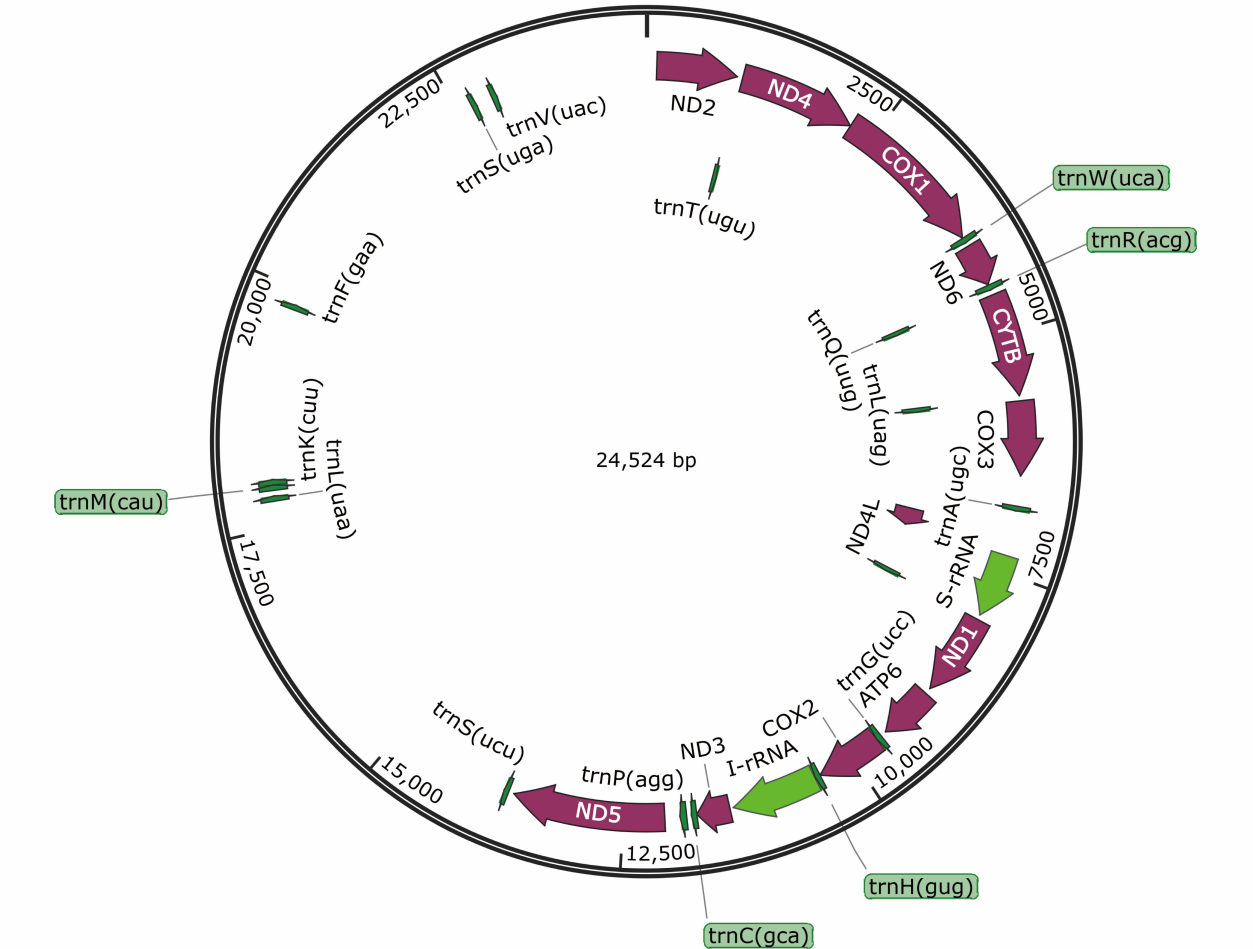
Annotated map of the intact mitochondrial genome of *M. muris*. The mitochondrial genome consists of protein-coding genes (plums), tRNAs (green), and rRNAs (light green). The inferred gene boundaries for these genes are shown in Table 4.

**Fig 4:**
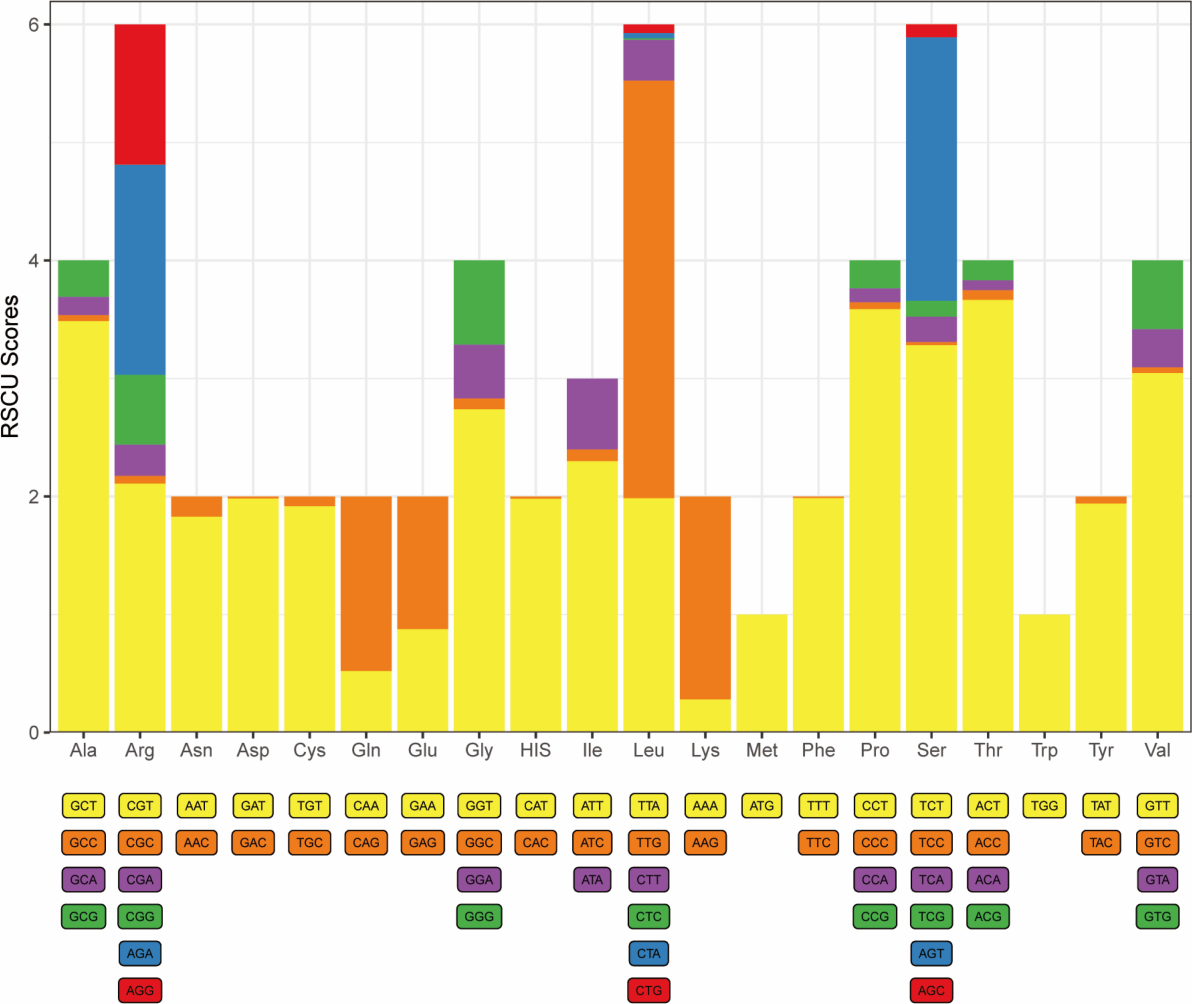
Bar graph of the codon-biased column of *M. muris*. The abscissa indicates the amino acid type of codon translation, and the ordinate represents the codon bias score calculated for that amino acid. The higher the score, the more types of codons, and the more active the evolutionary variation of the genes in the genome.

The mitochondrial genome of *M. muris* was sequenced, and the genome length of *M. muris* was determined to be 24,524 base pairs (GenBank ID: PP584052). The mitochondrial genome map showed a typical nematode genome structure as a circular double-stranded DNA molecule (Fig. 3). By sequencing, assembling, and annotating the mitochondria, the mitochondrial group of *M. muris* was found to contain 12 PCGs (ND4L, CYTB, ND1-6, *cox 1-3*, ATP6), 18 tRNA genes, 2 rRNA genes (s-rRNA and I-rRNA), which was a total of 32 genes all encoded on the same strand (Table 4). To indicate the frequency of codon use, the relative synonymous codon use (RSCU) values of the mitochondrial genome of *M. muris* were visualized (Fig. 4; Table 5). The analysis revealed the most commonly used codons to be TTT, GTT, TTG, TTA, and ATT; thus, phenylalanine (Phe), valine (Val), isoleucine, and leucine (Leu) were the most commonly used amino acids. In addition, RSCU analysis revealed that more AT than GC was used at the third codon position. The frequency of codon use indicates the preference of the nucleotide AT in *M. muris*.

**TABLE 4:** Gene length and position in the mitochondria genomes of *M. muris*

Gene	Type	Gene num	Exon num	Location	Gene length
ND2	CDS	1	1	110–955:+^*a*^	864 bp
trnT(ugu)	tRNA	1	1	957–1,011:+	55 bp
ND4	CDS	1	1	1,011–2,240:+	1,230 bp
*cox 1*	CDS	1	1	2,242–3,897:+	1,656 bp
trnW(uca)	tRNA	1	1	3,903–3,960:+	58 bp
ND6	CDS	1	1	4,002–4,450:+	449 bp
trnR(acg)	tRNA	1	1	4,459–4,513:+	55 bp
trnQ(uug)	tRNA	1	1	4,515–4,568:+	54 bp
CYTB	CDS	1	1	4,568–5,653:+	1,086 bp
trnL1(uag)	tRNA	1	1	5,655–5,709:+	55 bp
*cox 3*	CDS	1	1	5,709–6,488:+	780 bp
trnA(ugc)	tRNA	1	1	6,804–6,855:+	52 bp
ND4L	CDS	1	1	7,090–7,332:+	243 bp
s-rRNA	rRNA	1	1	7,326–8,000:+	675 bp
trnY(gua)	tRNA	1	1	8,001–8,055:+	55 bp
ND1	CDS	1	1	8,053–8,926:+	874 bp
ATP6	CDS	1	1	8,992–9,570:+	579 bp
trnG(ucc)	tRNA	1	1	9,632–9,688:+	57 bp
*cox 2*	CDS	1	1	9,689–10,387:+	699 bp
trnH(gug)	tRNA	1	1	10,388–10,442:+	55 bp
I-rRNA	rRNA	1	1	10,448–11,351:+	904 bp
ND3	CDS	1	1	11,389–11,737:+	349 bp
trnC(gca)	tRNA	1	1	11,738–11,793:+	56 bp
trnP(agg)	tRNA	1	1	11,845–11,913:+	69 bp
ND5	CDS	1	1	12,072–13,667:+	1596 bp
trnS1(ucu)	tRNA	1	1	13,724–13,776:+	53 bp
trnL2(uaa)	tRNA	1	1	17,776–17,829:+	54 bp
trnM(cau)	tRNA	1	1	17,888–17,9442:+	55 bp
trnK(cuu)	tRNA	1	1	17,943–17,999:+	57 bp
trnF(gaa)	tRNA	1	1	19,788–19,842:+	55 bp
trnS2(uga)	tRNA	1	1	22,652–22,703:+	52 bp
trnV(uac)	tRNA	1	1	22,878–22,931:+	54 bp

^*a*^ ":+" indicates that the transcription direction is positive.

**TABLE 5:** RSCU in the mitochondrial genome of *M. muris^a^*

Amino acid	Codon	Usage frequency	RSCU
Phe	TTT	1,368	1.987
	TTC	112	0.013
Ser	TCT	161	3.283
	TCC	28	0.027
	TCA	55	0.215
	TCG	26	0.135
	AGT	183	2.233
	AGC	18	0.108
Tyr	TAT	398	1.941
	TAC	55	0.059
Cys	TGT	286	1.918
	TGC	47	0.082
Trp	TGG	208	1
Leu	CTT	153	0.345
	CTC	20	0.014
	CTA	65	0.043
	CTG	51	0.072
	TTA	437	1.986
	TTG	463	3.54
Pro	CCT	46	3.588
	CCC	9	0.059
	CCA	6	0.118
	CCG	8	0.235
His	CAT	49	1.981
	CAC	3	0.019
Gln	CAA	21	0.522
	CAG	36	1.478
Arg	CGT	39	2.11
	CGC	1	0.066
	CGA	9	0.264
	CGG	23	0.593
	AGA	87	1.78
	AGG	94	1.187
Ile	ATT	437	2.3
	ATC	46	0.1
	ATA	180	0.6
Met	ATG	145	1
Thr	ACT	88	3.667
	ACC	15	0.083
	ACA	29	0.083
	ACG	10	0.167
Asn	AAT	225	1.829
	AAC	28	0.171
Lys	AAA	117	0.282
	AAG	100	1.718
Val	GTT	470	3.048
	GTC	46	0.048
	GTA	146	0.323
	GTG	113	0.581
Ala	GCT	68	3.487
	GCC	16	0.051
	GCA	27	0.154
	GCG	27	0.308
Asp	GAT	121	1.983
	GAC	30	0.017
Glu	GAA	66	0.877
	GAG	88	1.123
Gly	GGT	226	2.74
	GGC	41	0.091
	GGA	66	0.457
	GGG	79	0.712
*	TGA*	163	0.29
	TAG*	165	0.3
	TAA*	230	0.41

^*a*^ *Stop codon.

### Ka/Ks nonsynonymous mutation ratio

**Fig 5:**
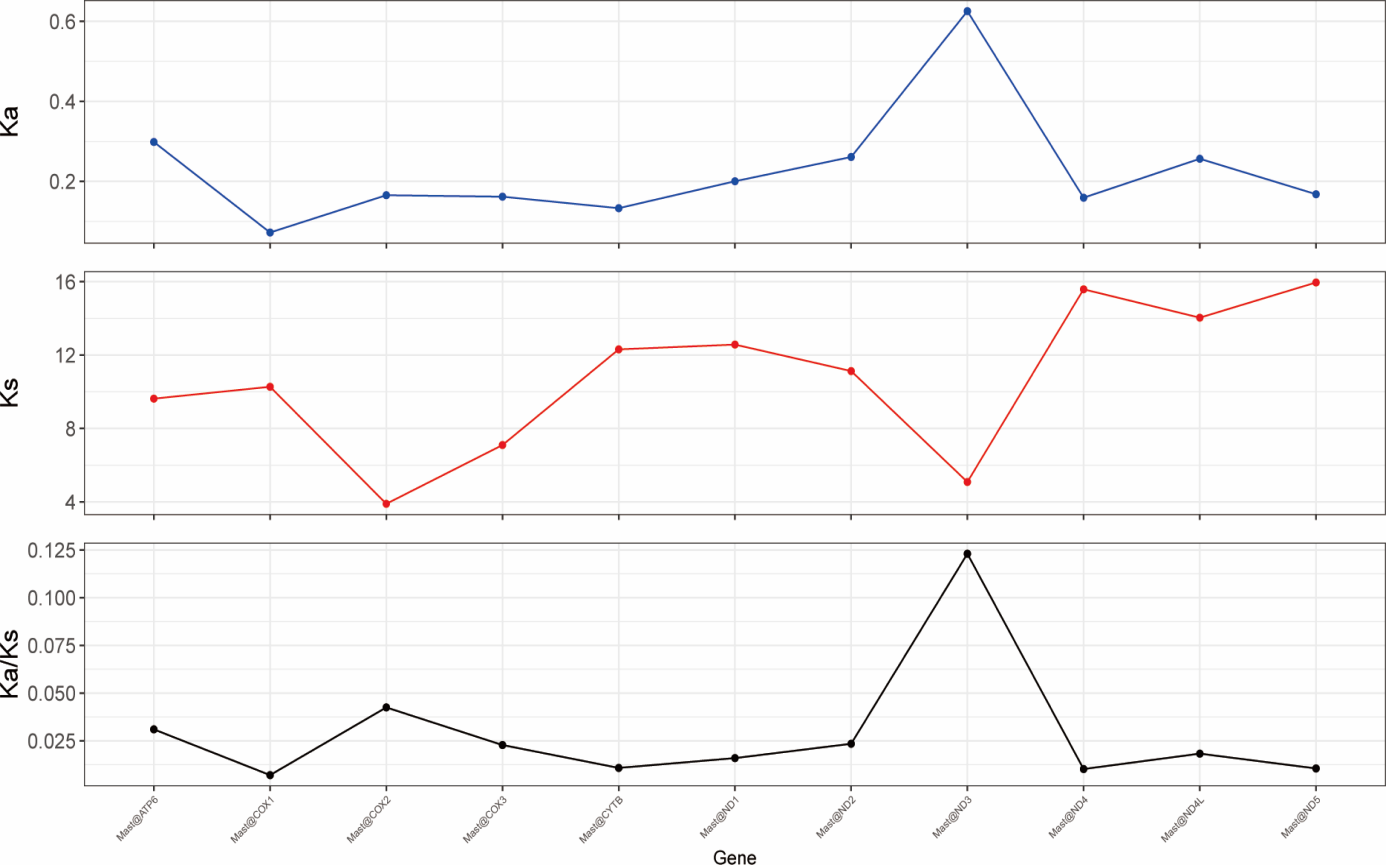
Scatter plot of Ka/Ks distribution. The abscissa represents the genes in order, and the ordinate represents Ka, Ks, and Ka/Ks ratios from top to bottom. The Ka/Ks ratios for these genes are shown in Table 6.

Ka/Ks, also known as the ratio between the number of nonsynonymous substitution sites (Ka) and the number of synonymous substitution sites (Ks), can determine whether there is selective pressure acting on PCGs. Ka/Ks can be used as an important marker in estimating the rate of evolution. The Ka/Ks values of all PCGs were < 1, suggesting that the genes were evolutionarily conserved in the mitochondrial genome (Fig. 5). ND6 had the highest Ka/Ks value, followed by *cox 2* and ATP6, whereas *cox 1* had the lowest Ka/Ks value (Ka/Ks = 0.007), indicating a low evolution rate. These results indicated the strong purification selection and evolutionary conservatism of the *cox 1* gene, which can be used as an important marker to identify the genetic relationship between species. In contrast, ND6 has the highest Ka/Ks value of 0.293, exhibiting a faster evolution rate and lower selection pressure in PCGs, whereas PCGs have a relatively weak selection for purification.

**TABLE 6:** The evolutionary rate of each PCGs in the mitogenomes of 11 gomphocerine species

Sequence	Ka	Ks	Ka/Ks
ND2	0.261056	11.1297	0.023456
ND4	0.159252	15.5859	0.010218
*cox 1*	0.072282	10.2727	0.007036
CYTB	0.133081	12.3114	0.01081
*cox 3*	0.16183	7.09841	0.022798
ND4L	0.256599	14.0384	0.018278
ND1	0.200389	12.573	0.015938
ATP6	0.298518	9.62618	0.031011
*cox 2*	0.165518	3.8947	0.042498
ND3	0.625472	5.08505	0.123002
ND5	0.167971	15.9575	0.010526

### Phylogenetic relationship

**Fig 6:**
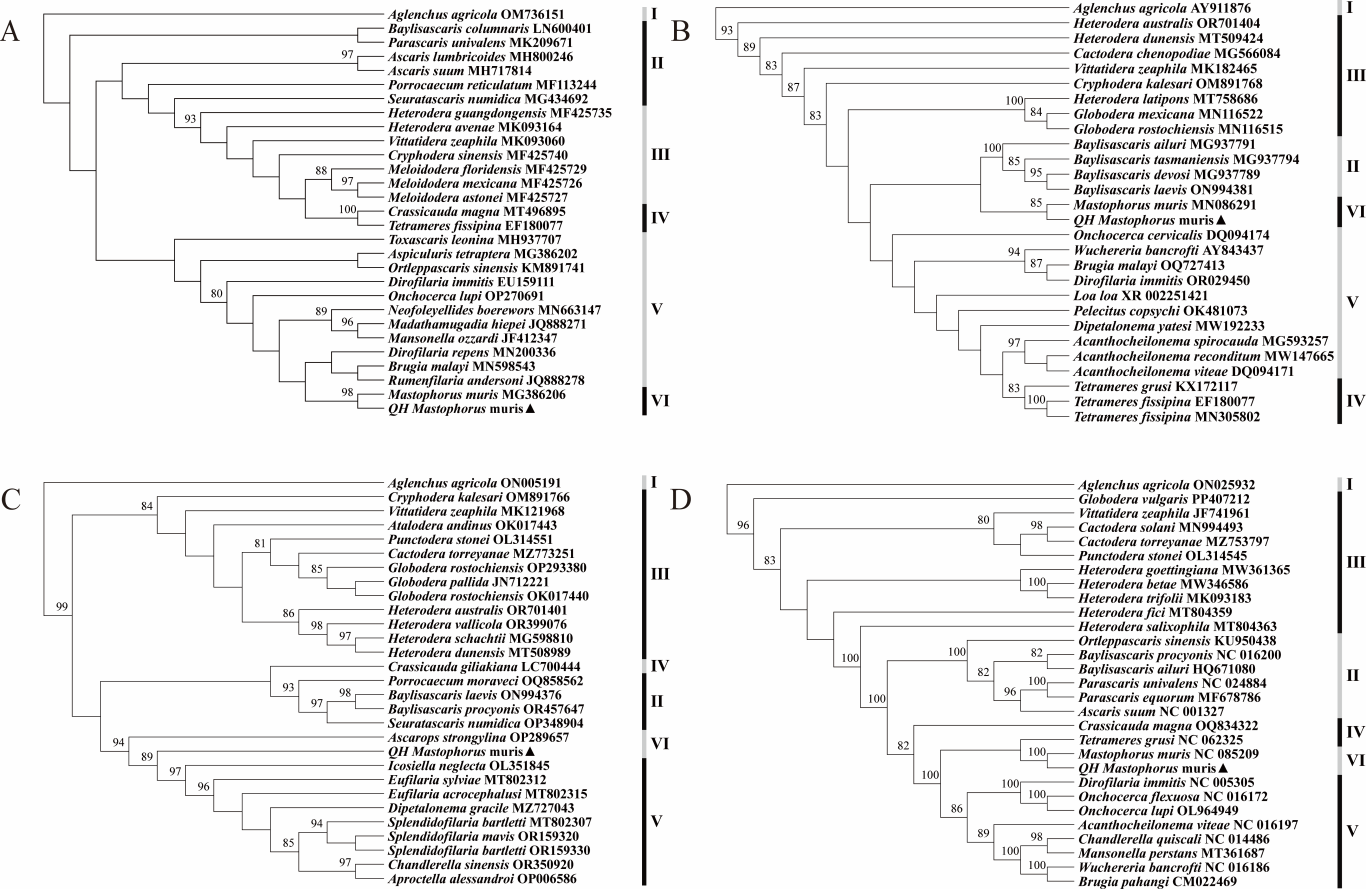
Based on the nucleotide sequences of mitochondrial *cox 1 *(A), 18S rRNA (**B**), 28S rRNA (**C**), and 12PCGs (**D**), the phylogenetic relationship between *M. muris* and other nematodes was inferred by maximum likelihood methodology approaches. Black triangle indicates the *M. muris* obtained from *Neodon fuscus* in this study. Alternating black and gray bands show diferent genera in the NCBI classifcation, and Roman numerals to the right of the bands indicate outgroups and different genus names. I: *Aglenchus agricola* was selected as outgroups, II: Ascarididae, III: Heteroderidae, IV: Tetrameridae, V: Onchocercidae, and VI: Spirocercidae. Numbers along branches are bootstrap support (BS) values, and only values of BS ≥ 80% are shown at the nodes.

**Fig 7:**
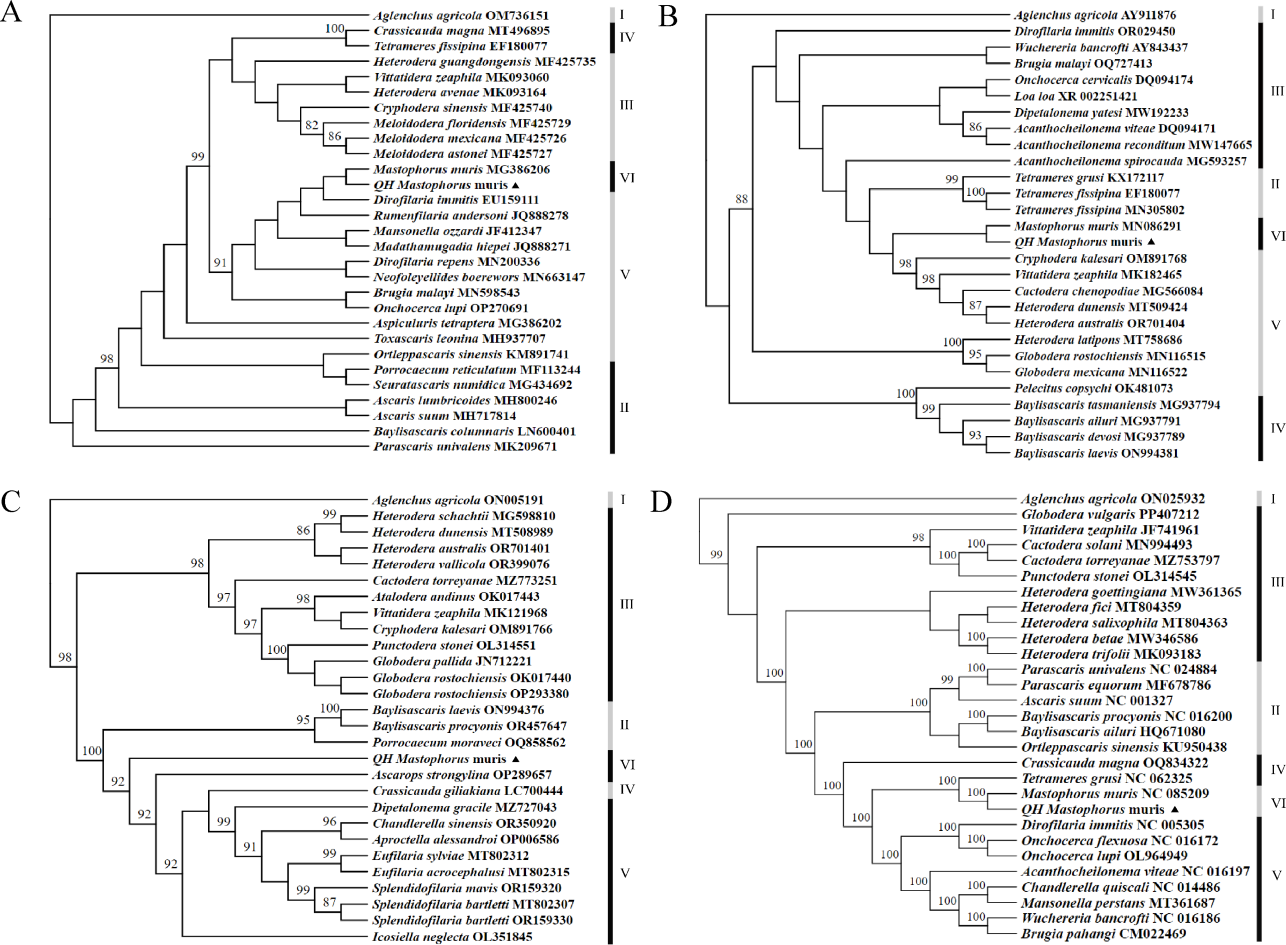
Validation of the phylogenetic relationships of *M. muris* and other nematodes based on the nucleotide sequences of mitochondrial *cox 1* (A), 18S rRNA (B), 28S rRNA (C), and 12PCGs (D) using Bayesian inference methodology approaches. Black triangle indicates the *M. muris* obtained from *Neodon fuscus* in this study. Alternating black and gray bands show diferent genera in the NCBI classifcation, and Roman numerals to the right of the bands indicate outgroups and different genus names. I: *Aglenchus agricola* was selected as outgroups, II: Ascarididae, III: Heteroderidae, IV: Tetrameridae, V: Onchocercidae, and VI: Spirocercidae. Only values of BS ≥ 80% are shown at the nodes.

Two different methods were used to analyze the relationship of *M. muris* with other genera and families using *cox 1* fragments, 18S rRNA, 28S rRNA, and 12 PCGs. Although different methods and data were used, the phylogenetic tree revealed that the sample is far from the clade in which other nematodes have been reported in GenBank; however, it was in the same clade and most closely related to *M. muris*, which belongs to the gastric parasitic nematode. Furthermore, *M. muris* was located in the same clade as the Onchocercidae family, suggesting its close relationship with the Onchocercidae family. The results are shown in Fig. 6 and 7.

### Divergence time analysis

**Fig 8:**
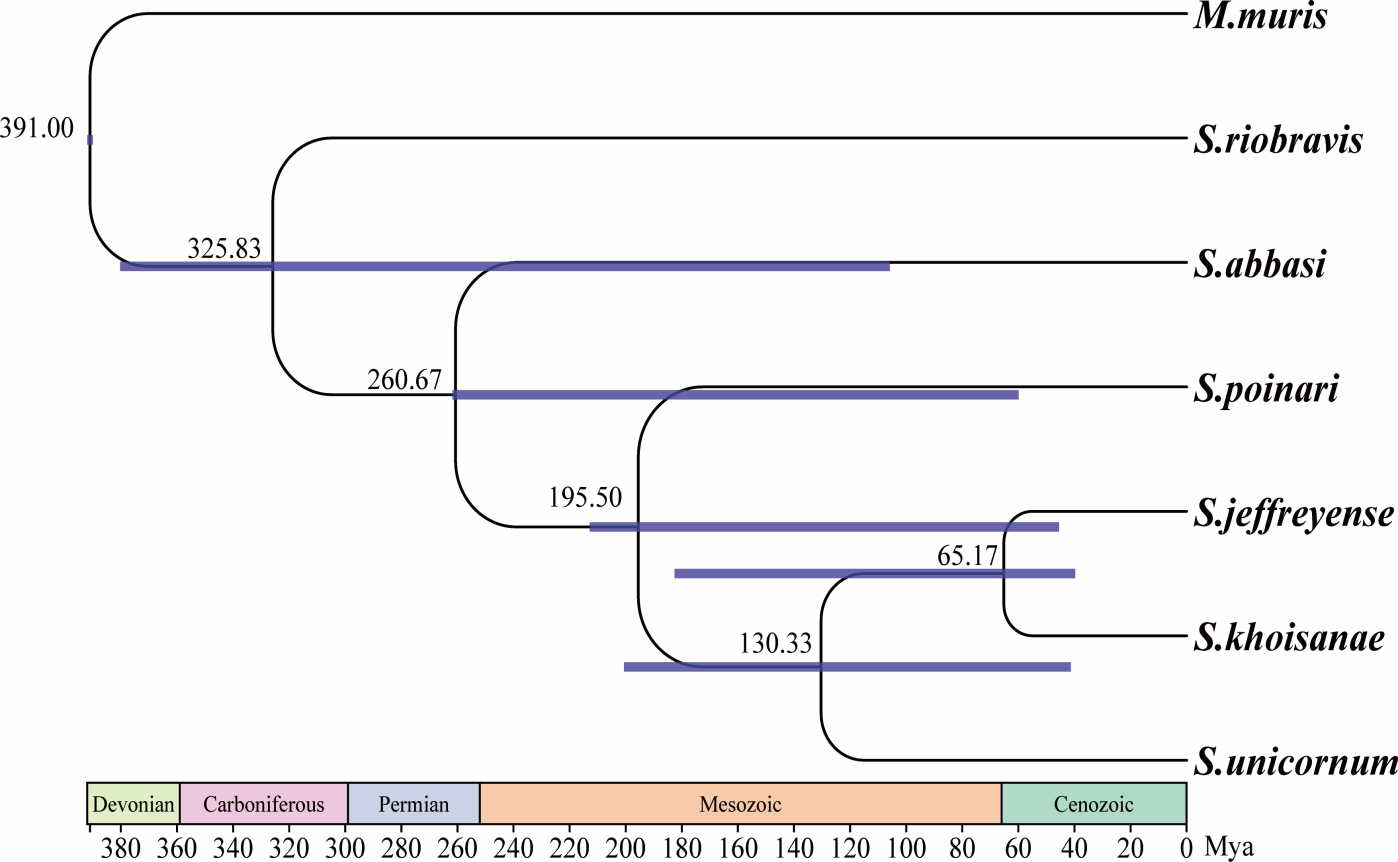
The differentiation time between *M. muris* and Steinernematidae family was constructed based on mitochondrial *cox 1* sequence. The number on the node indicates the divergence time between the two lineages. The blue bar indicates the time interval between the CA 95% highest probability density.

Analysis of the differentiation time of the mitochondrial PCGs of *M. muris* and the Steinernematidae family revealed the differentiation time to be 391 Mya (Fig. 8).

## DISCUSSION

The pharynx, teeth, and lip structures change during the development of *M. muris* (2). *M. muris* has been classified by Chiwood in the genus *Mastophorus* (36). Some scholars also believe that the *Protospirura* and *Mastophorus* genera are similar in morphology (12, 37). *M. muris* cannot be accurately identified using only morphological methods. Therefore, *M. muris* was identified at the molecular level in this study.

High-throughput sequencing technology was used to analyze the mitochondrial genome of *M. muris*. A total of 5,983,878,900 base pairs of raw data were obtained in this sequencing, and 5,728,247,284 base pairs of high-quality clean data were obtained after filtering. This finding indicated the sequencing results to be reliable and provided a guarantee for subsequent related analysis. The quality control results for the sequencing showed that Q20 and Q30 reached 97.50% and 93.40%, respectively, and the mitochondrial genome size of *M. muris* was 24,524 base pairs. The base distribution showed obvious AT bias, and the AT content was significantly higher than GC content (27%), which was consistent with the results from previous studies on nematodes (38–40).

The whole mitochondrial genome of *M. muris* in *Neodon fuscus* was obtained for the QTP. Like most other nematodes, the ATP8 gene was not found in the mitochondrial genome of *M. muris*, consistent with that in other nematodes (except for *Trichinella*) (41–45). However, *M. muris* lacked four tRNAs that are present in other nematodes (46, 47). The study showed that the number of tRNAs depends not only on the mt-genetic code but also on the evolutionary history of the taxon (48). These may be unknown factors related to the ability of mtDNA to acquire/lose genetic material.

Codon use bias (CUB) refers to the fact that different codons exhibit different frequencies of use in the genome (49, 50). Some studies have found little correlation between codon use bias and species evolution in the mitochondrial genome. Furthermore, there is no evidence that the choice of synonymous codon use affects the pattern of protein evolution in organisms (51). Thus, a detailed analysis of mitochondrial genome codon use will provide a better understanding of the evolutionary relationship in nematodes (50). Additionally, studying CUB is essential in understanding genome structure, function, and evolutionary processes (52). However, no systematic studies have been reported on codon use of *M. muris*. This study found that Phe was one of the most commonly used amino acids in the mitochondrial PCGs of *M. muris*, followed by Val and Leu. This finding was consistent with that reported for nematode mitochondria (53–55). CUB is influenced by several factors, including genetics, environmental factors, and evolutionary pressures (56–59). A recent study found that mutation stress may play a crucial role in shaping the use of codons (60). We speculated that the CUB composition of *M. muris* might be related to the unique environment of the QTP.

The mitochondrial genome was used in this study to analyze the phylogenetic relationship between *M. muris* derived from *Neodon fuscus* and other nematode data downloaded from NCBI. *M. muris* was clearly isolated from the other nematode branches in the phylogenetic tree constructed for the mitochondrial genome. Furthermore, the phylogenetic tree constructed using the highly conserved *cox 1* gene as a marker for the mitochondrial genome (61) showed the *M. muris* sequence from *Neodon fuscus* also formed a small branch in the phylogenetic tree. Analysis of the phylogenetic tree revealed that *M. muris* was located in the same clade as the Onchocercidae family, suggesting its close relation with the Onchocercidae family. However, we did not immediately make any systemic changes to *M. muris*, and preliminary phylogenetic results must be used with caution. Therefore, a deeper study of the phylogeny of *M. muris* is warranted.

Analysis of the divergence time of the mitochondrial PCG of *M. muris* showed a divergence time of 391 Mya between *M. muris* and the Steinernematidae family (Fig. 8). However, very limited data were obtained due to the lack of relevant fossils. It was difficult to understand their evolutionary origins from these clues, and more data and investigations are needed to provide further insights. Species diversification was mainly caused by biotic and abiotic factors (62). This study found that *M. muris* was almost confined to the Mediterranean continent (63) and that the initial collision of the Arab Eurasian continent was limited to before the Eocene epoch (> 56 Mya) (64). The abrupt pulse of the massive terrigenous sediment influx and deposition has important implications on the Cenozoic evolution of the Mediterranean during 24-21 Mya (65). Moreover, the accelerated uplift of the QTP occurred during the Miocene period (66, 67). Therefore, we speculated that *M. muris* from the QTP might have originated from the Mediterranean.

Many studies have found that *M. muris* can parasitize water rats, badgers (*Meles meles*), and other wild, carnivorous animals (6, 63, 68). *M. meles* is found in China, the United Kingdom, Hungary, Spain, and Italy (69, 70). This species consumes complex foods, exhibits tolerance and resistance to certain pathogens, and can be considered ecosystem engineers and a relevant natural factor in shaping species diversity (70, 71). We speculated that it might have been the mutual transmission between *M. meles* in the Mediterranean that transmitted *M. muris* to the QTP. From a morphological perspective, *M. muris* might have emerged to adapt to the newly emerging species in the Muridae and Microtidae families (2), as depicted in this study. Therefore, we speculated that biological factors might play a role in the formation or transmission of *M. muris* to some extent. Although the reasons for the formation of *M. muris* are unknown, this study showed that speciation was often the result of a genetic drift or adaptive differentiation between geographically distinct populations for most free-living organisms (62). Therefore, it could be speculated that the evolution of *M. muris* may depend on the mutual transmission between *M. meles.*

### Conclusions

The whole mitochondrial genomes of *M. muris* in the QTP were sequenced in this study. Analysis of the phylogenetic tree revealed that *M. muris* and the Onchocercidae family were located in the same branch and that they were closely related. Divergence time results showed that the divergence time of *M. muris* and the Steinernematidae family was 391 Mya. We speculated that *M. muris* in the QTP might have originated from the Mediterranean and might have been a result of the mutual transmission between *M. meles* in the Mediterranean, transmitting *M. muris* to the QTP. Our findings enrich the theoretical basis for the molecular identification and genetic research of *M. muris* and provide a scientific basis for its molecular and epidemiological investigation. In addition to traditional morphological identification, the use of molecular biology methods has supplemented our understanding of *M. muris* to a certain extent. The accuracy of the identification of *M. muris* species has improved and provides a basis for future phylogenetic and differentiation studies of *M. muris* from the QTP.

## Data Availability

In this study, sequence data generated are available in the GenBank with the accession numbers PP501782, PP140777, PP339708, and PP584052.

## SUPPLEMENTAL MATERIAL

Table S1 spectrum.03067-24-s0001.xlsx More detailed information for samples listed in Fig. 6 and 7.

ASM does not own the copyrights to Supplemental Material that may be linked to, or accessed through, an article. The authors have granted ASM a non-exclusive, world-wide license to publish the Supplemental Material files. Please contact the corresponding author directly for reuse.
